# Long-term survival and quality of life analysis in head and neck cancer survivors: An observational, cross-sectional study

**DOI:** 10.4317/jced.62744

**Published:** 2025-10-17

**Authors:** Karisa Lorena Freitas Limas, Giulianna Aparecida Vieira Barreto, Thinali Sousa Dantas, Paulo Goberlanio Barros Silva, Jose Fernando Bastos de Moura

**Affiliations:** 1 Oncology Department in Cancer Institute of Ceará, Brazil; 2 Christus University Center, Ceara, Brazil

## Abstract

**Background:**

Head and neck cancer is a public health problem, and its treatment involves surgery and/or radio/chemotherapy. These procedures reduce the quality of life. To analyze survival in patients with head and neck cancer and survivors, the quality of life over 10 years.

**Material and Methods:**

An observational, cross-sectional, retrospective study was carried out on 460 medical records of patients diagnosed with head and neck cancer and treated at a referral hospital. Clinical pathological data were expressed in absolute and percentage frequencies, and the median survival time was calculated with their 95% confidence intervals using Kaplan-Meier curves. The curves were compared using the Log-Rank Mantel-Cox test and Cox regression. In survivors, we applied a quality of life (QoL) questionnaire where means and standard deviations of quality of life domains were calculated and compared using the Friedman/Dunn test and correlated with time after discharge using Spearman's correlation. Quality of life, classified as low or high with an average of 80 points, was associated with other clinical-pathological characteristics during the period of diagnosis using Pearson's chi-square test. All analyses performed adopted a 95% CI in the SPSS software. v20.0 for Windows.

**Results:**

The medical records were analyzed; most of them were male, with a mean age of 61 years and a history of smoking and alcoholism. The most frequent staging was stage IV (T4, N2, M0). The most frequent location was the oropharynx, followed by the mouth. The median overall survival was 26.7 months (95% CI = 19.8-33.7). Of the 173 living patients, 41 (23.7%) responded to the questionnaire. Of the main problems related to QoL, swallowing (n = 10; 25.6%) was the most important, followed by chewing (n = 9; 28.1%) and saliva (n = 12; 42.9%). When QoL was associated with variables, it was observed that smoking (p = 0.014), alcoholism (p = 0.029), and time since discharge (p = 0.044) were associated with worse quality of life.

**Conclusions:**

Cancer treatment has numerous consequences for the quality of life of patients who have survived head and neck cancer. Further studies and differentiated plans are needed for the rehabilitation of these patients.

## Introduction

Head and neck cancer is a heterogeneous group of malignant neoplasms that can affect the head and neck structures, including the mouth (lips, gums, palate, tongue, and floor), pharynx, larynx, and other regions. It is more common on the lateral border of the tongue and the floor of the mouth, with a predilection for males over 40 years of age. Oral lesions, cervical nodules or tumors, persistent hoarseness or change in voice, difficulty speaking or swallowing, facial or neck pain, nosebleeds or bleeding in the mouth, unexplained weight loss, and difficulty breathing should be evaluated by a specialist. The therapeutic delay has already shown an increase in patient suffering due to loss of functions and mutilations, in addition to increasing the cost of treatment, leading to social rejection and poor quality of survival (1) . Staging classification aims to assess prognosis, plan treatment, evaluate results, and exchange information between professionals and research institutions (2). Tumor stage is defined according to the WHO proposal: T is related to tumor size, N indicates lymph node involvement, and M represents distant metastases, considering stage I, stage II, stage III, or stage IV, covering stages IVA, IVB, and IVC. Survival is determined based on the difference between the date of initiation of treatment and the date of death (3). Treatment usually consists of surgery combined with chemotherapy and radiotherapy, depending on the extent and degree of involvement of the lesion. As a result of oncological treatment, if treated with surgery, oral mutilations may occur; if treated with radiotherapy or chemotherapy, they may present manifestations of toxicity such as xerostomia, hyposalivation, changes intTaste, mucositis, and bone necrosis. These manifestations are usually quite symptomatic, cause severe pain, impair the patient's nutrition and speech, significantly worsening the quality of life, and increase the costs of treatment. Treatment can cause significant changes in the affected individuals' vital functions related to feeding, communication, and social interaction (4). Early-stage lesions always have a better prognosis and high cure rates. Therefore, the dentist must establish preventive, early diagnosis, and therapeutic measures to minimize these problems and monitor patients with the multidisciplinary team to humanize care and improve the patient's quality of life. Neck cancer is undoubtedly related to a decrease in quality of life. After diagnosis, treatment often results in a deterioration of essential functions such as chewing, breathing, salivation, swallowing, and speech (5 - 8). Given this context, long-term research was necessary to outline a profile of how head and neck cancer survivors live, what their needs are, what their survival rate is, and what their quality of life is like. The aim was to understand the real impact of the disease and its treatment on these people's lives.

## Material and Methods

- Study design and sampling outline This is an observational, cross-sectional, and retrospective study. This study was divided into 2 phases. In phase 1, a retrospective case study was carried out, where medical records of patients with head and neck cancer treated at a referral hospital in Mossoró/RN from January 2011 to December 2021 were retrieved. The clinical and pathological data of the patients were collected, in addition to a query in the death information system (SIM). The date of the first treatment and the date of death were collected for survival analysis, and the contacts of surviving patients or those who did not appear as deceased in the SIM were collected so that the researcher could contact them by telephone. In phase 2, the patients from the previous series who survived and were located were then invited to participate in the research by telephone contact. At that time, the Free and Informed Consent Form-TCLE was sent via WhatsApp. Once accepted, the quality of life questionnaire was sent, which was prepared in Google Forms and sent individually and also answered via WhatsApp. This project was approved by the Technical Committee of the Hospital Liga Mossoroense de Estudos e Combate ao Câncer and by the Ethics Committee of the Instituto do Câncer do Ceará, following the research ethics regulations of Law 466/12, which governs research ethics precepts in Brazil. The STROBE Checklist guided this work to ensure compliance with international parameters for conducting observational studies. - Inclusion, exclusion, and withdrawal criteria Patients with a history of malignant tumors in the head and neck region who were treated at the reference hospital between January 2011 and December 2021 were included in the study. Patients with a history of neurological disease or cognitive impairment that could interfere with the interpretation and response to the informed consent form and the quality of life questionnaire were excluded. Patients who withdrew from the study during the questionnaire were also excluded. Those who could not be contacted were considered survivors until the date of the last consultation. - Quality of life analysis Quality of life was analyzed using the UW-QOL questionnaire, which was validated, for use in oncology patients with head and neck cancer. It is in its fourth version and contains 12 questions related to specific domains or functions of the head and neck region, such as activity, recreation, pain, mood, and anxiety. In the UW-QOL questionnaire, specific domains such as pain, mood, and anxiety were investigated. The questionnaire, created in Google Forms, was sent individually via WhatsApp and then returned to the researcher, who archived it in an Excel spreadsheet. In addition, the following data were extracted from the medical records: sex, age, tumor location, histopathological diagnosis, clinical stage of the tumor, staging, family history, date of last consultation, date of diagnosis, date of start of treatment, habits (such as smoking and alcohol consumption), and date of death. - Statistical analysis The clinical-pathological data were expressed as absolute and percentage frequencies, and the median survival time was calculated along with its 95% confidence intervals using Kaplan-Meier curves. The curves were compared using the Log-Rank Mantel-Cox test and Cox regression. Means and standard deviations of the quality of life domains were calculated for the surviving patients, compared using the Friedman/Dunn test, and correlated with time after discharge using Spearman's correlation. Quality of life was categorized as low or high using a cutoff of 80 points and associated with other clinical-pathological characteristics at the time of diagnosis using Pearson's chi-square test. All analyses were performed using a 95% confidence interval using SPSS v20.0 for Windows software.

## Results

- Clinical and sociodemographic profile of oncology patients with head and neck cancer Of the 460 patients in the study, the majority were male (n=348, 75.7%), with a mean age of 61.53±14.40 years ranging from 0-102 years, with the majority being in the age group of 51-70 years. Most patients reported a history of smoking (n=276, 60.0%) and alcoholism (n=247, 53.7%). The most frequent stage was stage IV (n=174, 54.4%), with the majority being T4 (n=147, 44.5%), N2 (n=134, 40.6%), and M0 (n=24, 7.3%). The most frequent location was the oropharynx (n=135, 29.3%), followed by the tongue (n=106, 23.0%). The other places of the primary tumor are available in Table 1.

Table 1

**Figure 1:**
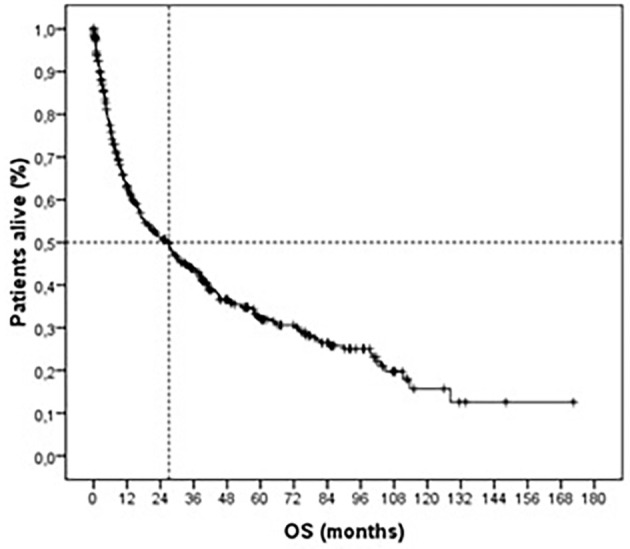
Kaplan-Meier curve of overall survival in head and neck cancer survivors.

The median overall survival of patients was 26.7 (95% CI = 19.8-33.7) months, with only 173 (37.6%) patients alive at a mean follow-up of 52.5±3.7 months (Fig. 1).

1

Primary factors associated with survival in oncology patients with head and neck cancer The main factors associated with survival were staging (p&lt;0.001), T (p&lt;0.001), N. (p&lt;0.001) and M (p p &lt;0.001) (Table 2).

Table 2

- Multivariate analysis of the most significant risk factors for mortality In multivariate analysis, N staging (p=0.004), followed by T staging (p=0.015), location in the mouth (p=0.040), and clinical stage (p=0.045) were the most critical risk factors for mortality (Table 3).

Table 3

**Figure 2:**
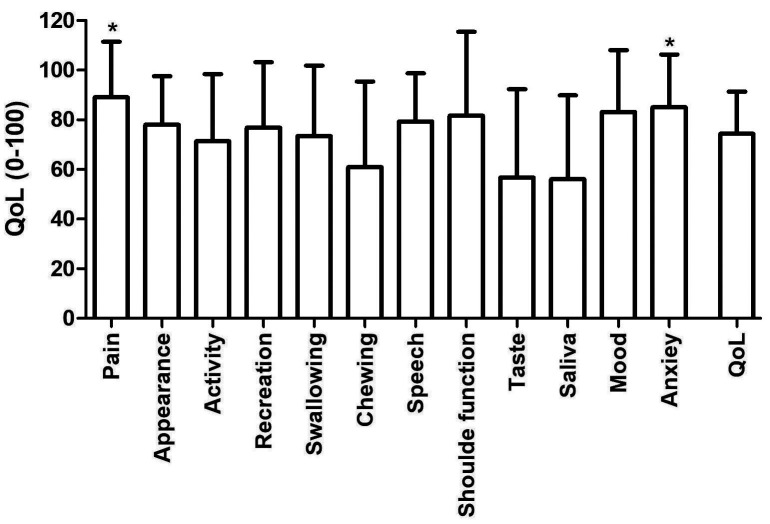
Quality-of-life analysis in head and neck cancer survivors.

Topic 4: Main problems associated with quality of life Of the 173 living patients, 41 responded to the questionnaire (23.7%). The mean QoL scores were (74.39±16.92). The Pain (89.02±22.39) and Anxiety (85.08±21.26) domains were significantly higher than the scores of Mood (83.13±24.93), Shoulder (81.68±33.74), Speech (79.33±19.45), Appearance (78.05±19.52), Amusement (76.83±26.45), Swallowing (73.43±28.47), Activity (71.34±27.13), Chewing (60.98±34.48), Taste (56.70±35.65) and Saliva (56.07±33.81) (p&lt;0.001) (Fig. 2).

2

Only 16 (39.0%) patients had scores higher than 80. Of the main problems related to QoL, swallowing was the most important (n=10, 25.6%). The second most important problem was chewing (n=9, 28.1%), and the third was saliva (n=12, 42.9%). When considering all of the issues mentioned, chewing and saliva were the most frequent (n=18, 18.2%), followed by swallowing (n=14, 14.1%) and speech (n=11, 11.1%) (Table 4).

Table 4

- Quality of life associated with other variables When QoL was associated with other variables, it was observed that a history of smoking (p=0.014) and alcoholism (p=0.029) and time since discharge (p=0.044) were associated with a worse quality of life (Table 5).

Table 5

**Figure 3:**
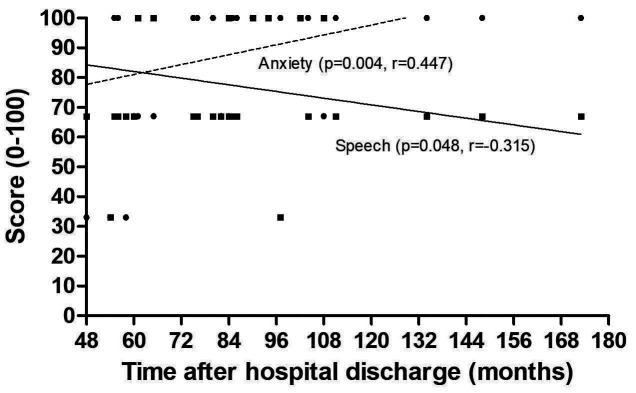
Correlation between time after treatment and levels of anxiety and speech. *p&lt;0.05, Spearman correlation.

The domains Pain (p=0.250), Appearance (p=0.510), Activity (p=0.978), Fun (p=0.697), Swallowing (p=0.390), Chewing (p=0.420), Shoulder (p=0.708), TasteTaste (p=0.670), Saliva (p=0.436), Mood (p=0.711), QoL Before CA (p=0.263), QoL Rel Health (p=0.756) and General QoL (p=0.846) did not show a significant correlation with the time after discharge. However, the Anxiety domain ( p=0.004, r=0.447) and Speech (p=0.048, r=-0.315) showed correlation significant with this variable (Fig. 3).

3

## Discussion

According to SESAP, in the state of RN, where Mossoró is located, between 2019 and 2023, 53,480 cases of cancer were registered, of which 23,284 (44%) were diagnosed in men and 30,196 (56%) in women, evidencing a disparity between the sexes. Similar findings were reported by Panov et al. (2021) (9), who highlighted that a combination of biological and socioeconomic factors influences gender disparities in oncological outcomes.

It is essential to highlight that, compared to 2019, there was an increase in the number of cancer cases in 2023, with the incidence rate rising to 287.8 cases per 100,000 inhabitants among men and 430.6 cases per 100,000 inhabitants among women. 2020 had the lowest incidence rate for both sexes in the historical series. However, it is essential to consider that 2020 was the peak of the COVID-19 pandemic, significantly impacting access to health services and mortality rates.

In our study, we observed an average of 46 patients with head and neck cancer (HNC) annually, totaling an average of 3.83 patients per month from 2011 to 2021. The sample profile was predominantly of male patients, with a mean age of 61 years, ranging from 0 to 102 years, with the majority concentrated in the age group of 51 to 70. These findings are similar to those found in the case series in Brazil.

The most frequent location of the cancers identified was the oropharynx, followed by the tongue, contrasting with previous studies and INCA statistics. Therefore, further investigations are needed to clarify whether cases of tongue cancer in the region are being underreported or undiagnosed or whether they represent a particularity of the service or the State. Research correlates these data with HPV vaccination, which began in the SUS only in 2014, and the population's habits are essential, considering that these factors directly contribute to the development of oropharyngeal cancer (10).

Most patients reported a history of smoking and alcohol consumption, corroborating the findings of Kfouri et al. (2018) (11) and Leite et al. (2021) (12), who highlight tobacco and alcohol as the main risk factors for head and neck cancer. The most common staging was T4, followed by N2 and M0, evidencing increasingly late diagnoses and treatments. In multivariate analysis, N and T staging, location in the mouth, and clinical stage were identified as the most relevant mortality factors, reinforcing the need for increasingly early diagnoses and treatments, as emphasized by Macedo et al. (2024) (13).

Our study indicated a median overall survival of 26.7 months, with only 173 patients alive in a median follow-up of 52.5 months. The main factors associated with survival were staging, T, N, and M. The data from our study corroborate the importance of early diagnosis in the survival of HNC patients, in line with the findings of Morais et al. (2022) (14) and Cramer et al. (2018) (15), who discuss the quality of life about the effects of treatment, late diagnosis, and patient survival. Our study was similar to that of patients with HNC in low- and middle-income regions such as South Asia, Southeast Asia, and sub-Saharan Africa, who have a survival rate of less than 26 months due to factors such as late diagnosis, limited access to advanced technologies, lack of oncologists, and inadequate medical infrastructure.

During our study, of these 41 patients who underwent quality of life assessments, we found that the absence of pain and anxiety levels were significantly higher than the levels of mood, shoulder pain, speech difficulties, appearance, fun, swallowing, activities, chewing, taste, and salivation during the assessment of their quality of life. The absence of pain and anxiety levels contributed positively to quality of life. On the other hand, functional domains such as chewing, taste, and salivation presented the worst scores, negatively impacting quality of life. This is understandable because chewing and taste are essential for eating and speech. The evaluation of these patients revealed significant impairments, resulting in a decrease in quality of life scores, which is in line with the results of Viana (2017) (16) , who indicated that stomatognathic functions related to speech were factors that compromised quality of life. In our study, only 16 patients presented scores higher than 80 (average). According to Rogers et al. (2002) (17) survival, the possibility of a cure, living as long as possible, and not feeling pain are priorities for patients post-treatment.

Patients' quality of life before cancer was considered slightly better (34.1%) and much better for only (14.6%). When asked about health-related quality of life, it was deemed suitable to (31.7%), perfect (17.1%), and excellent for only (2.4%) of the patients. In comparison, the overall quality of life in our study was classified as good (48.8%), perfect (14.1%), and excellent (7.3%), resulting in an average overall quality of life of 74.39 scores, where our average was 80 scores.

When associating QoL with other variables, it was observed that a history of smoking, alcoholism, and time since discharge were associated with a worse quality of life. We know that smoking compromises lung, cardiovascular, and immune function, increases complications after treatment, such as respiratory distress, fatigue, and reduced functional capacity, increases the risk of recurrence of new tumors, and this negatively impacts mental health, generates concerns about the future, and worsens quality of life. Cancer survivors who continue to smoke have lower levels of physical and mental quality of life compared to nonsmokers. Chronic alcohol consumption, on the other hand, impairs the immune system and metabolism, hindering recovery after cancer treatment and increasing the risk of infectious and inflammatory complications (16 , 17).

Among the main problems related to quality of life, swallowing was the most affected, followed by chewing and salivation. When considering all of the issues mentioned, chewing and salivation were the most frequent, followed by swallowing and speech, corroborating new data from Viana et al. (2017) (16), which demonstrated that functional problems in the stomatognathic system have a significant impact on the quality of life of patients with head and neck cancer.

Furthermore, tooth loss during pre-treatment mouth preparation to eliminate foci of oral infection can also contribute to poor chewing. According to a review by Sankar and Xu (2023) (18), up to 93% of head and neck cancer patients report xerostomia after radiotherapy, and more than 50% have significant difficulties in chewing and swallowing after surgery and/or radiotherapy. Another study published in the Journal of Clinical Oncology (19) highlights that these problems compromise quality of life, making rehabilitation with physiotherapy, speech therapy, and specific dental care essential.

In this study, some limitations were found since, in 10 years of treatment, therapeutic modalities change, medical records are often incomplete, it is challenging to locate patients due to changes in telephone contact, patients living in rural areas have complex telephone and internet signal, some patients who answered the phone did not provide any information for fear of responding, for fear of financial or cyber scams, other patients hung up because they did not know the researcher's number, many patients were lost to follow-up at the hospital, this limited a more in-depth analysis of long-term QoL, but even so, we were able to evaluate the survival of 41 patients over 10 years.

This study highlights the importance of tracking clinical factors and lifestyle habits, such as alcohol consumption and smoking, to improve both the quality of life and survival of patients with head and neck cancer. Early identification of these risk factors is crucial, as it can facilitate earlier diagnosis, thus reducing mortality associated with this pathology.

Furthermore, adopting a differentiated approach to these patients throughout the treatment process is essential, with special attention to the post-treatment period. Proper management of sequelae through rehabilitation programs is essential to minimize the negative consequences that cancer and its treatment can cause. Rehabilitation should focus on the recovery of basic functions, such as chewing, swallowing, and salivation, which are often severely compromised and significantly impact patients' quality of life.

Providing adequate conditions for these individuals to return to their daily activities, including eating, is crucial in restoring physical health and recovering self-esteem and social interaction. Being able to eat correctly and participate in family and social interactions contributes significantly to these patients' quality of life and emotional well-being.

Therefore, care for patients facing head and neck cancer should be multidisciplinary, involving not only health professionals but also psychological and social support. This integrated approach can provide a fuller reintegration into society, allowing patients to survive and live meaningful and productive lives with their self-esteem correctly restored.

## Figures and Tables

**Table 1:** Clinical and sociodemographic profile of oncology patients with head and neck cancer.

	N (%)
Total	460 (100.0%)
Sex	
Feminine	112(24.3%)
Masculine	348(75.7%)
Age (61.53±14.40; 0-102)	
Up to 50	82 (17.8%)
51-70	267(58.0%)
>70	111(24.1%)
Smoking	
No	184(40.0%)
Yes	276(60.0%)
Alcoholism	
No	213(46.3%)
Yes	247(53.7%)
Staging	
I	16 (5.0%)
II	46 (14.4%)
III	84 (26.3%)
IV	174(54.4%)
T	
T1	32 (9.7%)
T2	61 (18.5%)
T3	90 (27.3%)
T4	147(44.5%)
N	
N0	100(30.3%)
N1	43 (13.0%)
N2	134(40.6%)
N3	53 (16.1%)
M	
M0	305(92.7%)
M1	24 (7.3%)
Location	
Lip	9 (2.0%)
Language	106(23.0%)
Palate	28 (6.1%)
M jugal/retromolar	29 (6.3%)
Floor	36 (7.8%)
Gl salt larger	34 (7.4%)
Oropharynx	135(29.3%)
Nasopharynx	36 (7.8%)
Hypopharynx	47 (10.2%)

Caption: Data expressed as absolute and percentage frequency.Source: Survey Data (2024)

**Table 2:** Main factors associated with survival in oncology patients with head and neck cancer.

	SG (%)	SG (Months)	pp-Value
All	173/460(37.6%)	26.73(19.78-33.68)	-
Sex			
Feminine	49/112(43.8%)	27.40(9.31-45.49)	0.342
Masculine	124/348(35.6%)	26.67(19.09-34.25)	
Age			
Up to 50	34/82(41.5%)	28.17(16.04-40.30)	0.542
51-70	96/267(36.0%)	23.93(15.30-32.56)	
>70	43/111(38.7%)	27.83(11.86-43.80)	
Smoking			
No	85/184(46.2%)	38.17(25.60-50.74)	0.062
Yes	88/276(31.9%)	20.63(13.58-27.68)	
Alcoholism			
No	87/213(40.8%)	28.20(14.86-41.54)	0.735
Yes	86/247(34.8%)	23.33(16.08-30.58)	
Staging			
I/II	34/62(54.8%)	72.17(50.54-93.80)	<0.001
III	36/84(42.9%)	36.10(21.75-50.45)	
IV	44/174(25.3%)	13.80(9.39-18.21)	
T			
T1/T2	46/93(49.5%)	72.17(51.91-92.43)	<0.001
T3/T4	75/237(31.6%)	17.00(11.38-22.62)	
N			
N0	55/100(55.0%)	64.23(46.95-81.51)	<0.001
N+	66/230(28.7%)	16.17(9.68-22.66)	
M			
M0	116/305(38.0%)	29.27(19.85-38.69)	0.011
M1	5/24(20.8%)	13.80(5.75-21.85)	
Location			
Mouth	92/242(38.0%)	27.83(15.76-39.90)	0.681
Pharynx	81/218(37.2%)	23.93(15.13-32.73)	

Legend : *p<0.05, Log-Rank Mantel-Cox test. Overall survival was measured in median (95% confidence interval) months.Source: Research data (2024)

**Table 3:** Multivariate analysis of the most significant risk factors for mortality.

	Value	pp-aHR (95%CI)
Risk of death		
Sex	0.944	1.015 (0.673-1.530)
Age (>50)	0.003	1,440 (1,136-1,826)
Smoking	0.108	1.416 (0.926-2.166)
Alcoholism	0.616	0.892 (0.572-1.392)
Staging	*0.045	1.289 (1.005-1.652)
T	*0.015	1.593 (1.093-2.322)
N	*0.004	1,777 (1,197-2,639)
M	0.564	1.157 (0.704-1.902)
Location (mouth)	*0.040	1.356 (1.013-1.813)

Caption : *p<0.05, Cox regression; aHR = adjusted hazard risk-adjusted (95%CI)Source: Research data (2024)

**Table 4:** Main problems associated with quality of life.

	n (%)
QoL related issue (First)	
Swallowing	10 (25.6%)
Appearance	10 (25.6%)
Pain	5 (12.8%)
Chew	4 (10.3%)
Activity	3 (7.7%)
Anxiety	2 (5.1%)
Recreation	1 (2.6%)
Shoulder	1 (2.6%)
Palate	1 (2.6%)
Saliva	1 (2.6%)
He speaks	1 (2.6%)
Problem related to QoL (Second)	
Chew	9 (28.1%)
He speaks	6 (18.8%)
Saliva	5 (15.6%)
Swallowing	4 (12.5%)
Palate	4 (12.5%)
Shoulder	2 (6.3%)
Recreation	1 (3.1%)
Fun	1 (3.1%)
QoL related issue (third party)	
Saliva	12 (42.9%)
Chew	5 (17.9%)
He speaks	4 (14.3%)
Anxiety	4 (14.3%)
Shoulder	1 (3.6%)
Palate	1 (3.6%)
Humor	1 (3.6%)
QoL related issue (Any)	
Chew	18 (18.2%)
Saliva	18 (18.2%)
Swallowing	14 (14.1%)
He speaks	11 (11.1%)
Appearance	10 (10.1%)
Anxiety	6 (6.1%)
Palate	6 (6.1%)
Pain	5 (5.1%)
Shoulder	4 (4.0%)
Activity	3 (3.0%)
Recreation	2 (2.0%)
Fun	1 (1.0%)
Humor	1 (1.0%)
QoL Before CA	
Much worse	1 (2.4%)
A little worse	7 (17.1%)
More or less the same	13 (31.7%)
A little better	14 (34.1%)
Much better	6 (14.6%)
Health Rel QoL	
Bad	4 (9.8%)
Average	16 (39.0%)
Good	13 (31.7%)
Very good	7 (17.1%)
Excellent	1 (2.4%)
Overall QoL	
Bad	1 (2.4%)
Average	11 (26.8%)
Good	20 (48.8%)
Very good	6 (14.6%)
Excellent	3 (7.3%)

Caption: Data expressed as absolute and percentage frequency.Source: Research data (2024)

**Table 5:** Quality of life associated with other variables.

	QoL		p-
	Low	High	p-value
All	25(61.0%)	16(39.0%)	-
Sex			
Feminine	7 (28.0%)	5 (31.3%)	0.823
Masculine	18 (72.0%)	11 (68.8%)	
Age			
Up to 50	3 (12.0%)	3 (18.8%)	0.401
51-70	19 (76.0%)	9 (56.3%)	
>70	3 (12.0%)	4 (25.0%)	
Smoking			
No	6 (24.0%)	10 (62.5%)	0.014
Yes	19 (76.0%)*	6 (37.5%)	
Alcoholism			
No	7 (28.0%)	10 (62.5%)*	0.029
Yes	18 (72.0%)*	6 (37.5%)	
Staging			
I/II	8 (40.0%)	6 (50.0%)	0.226
III	5 (25.0%)	5 (41.7%)	
IV	7 (35.0%)	1 (8.3%)	
T			
T1/T2	11 (55.0%)	6 (50.0%)	0.784
T3/T4	9 (45.0%)	6 (50.0%)	
N			
N0	11 (55.0%)	7 (58.3%)	0.854
N+	9 (45.0%)	5 (41.7%)	
M			
M0	19 (95.0%)	12 (100.0%)	0.431
M1	1 (5.0%)	0 (0.0%)	
Location			
Mouth	17 (68.0%)	7 (43.8%)	0.124
Pharynx	8 (32.0%)	9 (56.3%)	
Time after discharge			
Up to 96 months	20 (80.0%)*	8 (50.0%)	0.044
>96 months	5 (20.0%)	8 (50.0%)*	

Legend : *p<0.05, Fisher’s exact or Pearson’s chi-square test (n, %).Source: Research data (2024)

## Data Availability

The datasets used and/or analyzed during the current study are available from the corresponding author.
